# Enhanced surveillance during a public health emergency in a resource-limited setting: Experience from a large dengue outbreak in Solomon Islands, 2016-17

**DOI:** 10.1371/journal.pone.0198487

**Published:** 2018-06-07

**Authors:** Adam T. Craig, Cynthia A. Joshua, Alison R. Sio, Bobby Teobasi, Alfred Dofai, Tenneth Dalipanda, Kate Hardie, John Kaldor, Anthony Kolbe

**Affiliations:** 1 The Kirby Institute, Faculty of Medicine, University of New South Wales, Sydney, Australia; 2 Solomon Islands Ministry of Health and Medical Services, Honiara, Solomon Islands; 3 Division of Pacific Technical Support, World Health Organization, Suva, Fiji; Instituut voor Tropische Geneeskunde, BELGIUM

## Abstract

Between August-2016 and April-2017, Solomon Islands experienced the largest and longest-running dengue outbreak on record in the country, with 12,329 suspected cases, 877 hospitalisations and 16 deaths. We conducted a retrospective review of related data and documents, and conducted key informant interviews to characterise the event and investigate the adaptability of syndromic surveillance for enhanced and expanded data collection during a public health emergency in a low resource country setting. While the outbreak quickly consumed available public and clinical resources, we found that authorities were able to scale up the conventional national syndrome-based early warning surveillance system to support the increased information demands during the event demonstrating the flexibility of the system and syndromic surveillance more broadly. Challenges in scaling up included upskilling and assisting staff with no previous experience of the tasks required; managing large volumes of data; maintaining data quality for the duration of the outbreak; harmonising routine and enhanced surveillance data and maintaining surveillance for other diseases; producing information optimally useful for response planning; and managing staff fatigue. Solomon Islands, along with other countries of the region remains vulnerable to outbreaks of dengue and other communicable diseases. Ensuring surveillance systems are robust and able to adapt to changing demands during emergencies should be a health protection priority.

## Introduction

**Fig 1:**
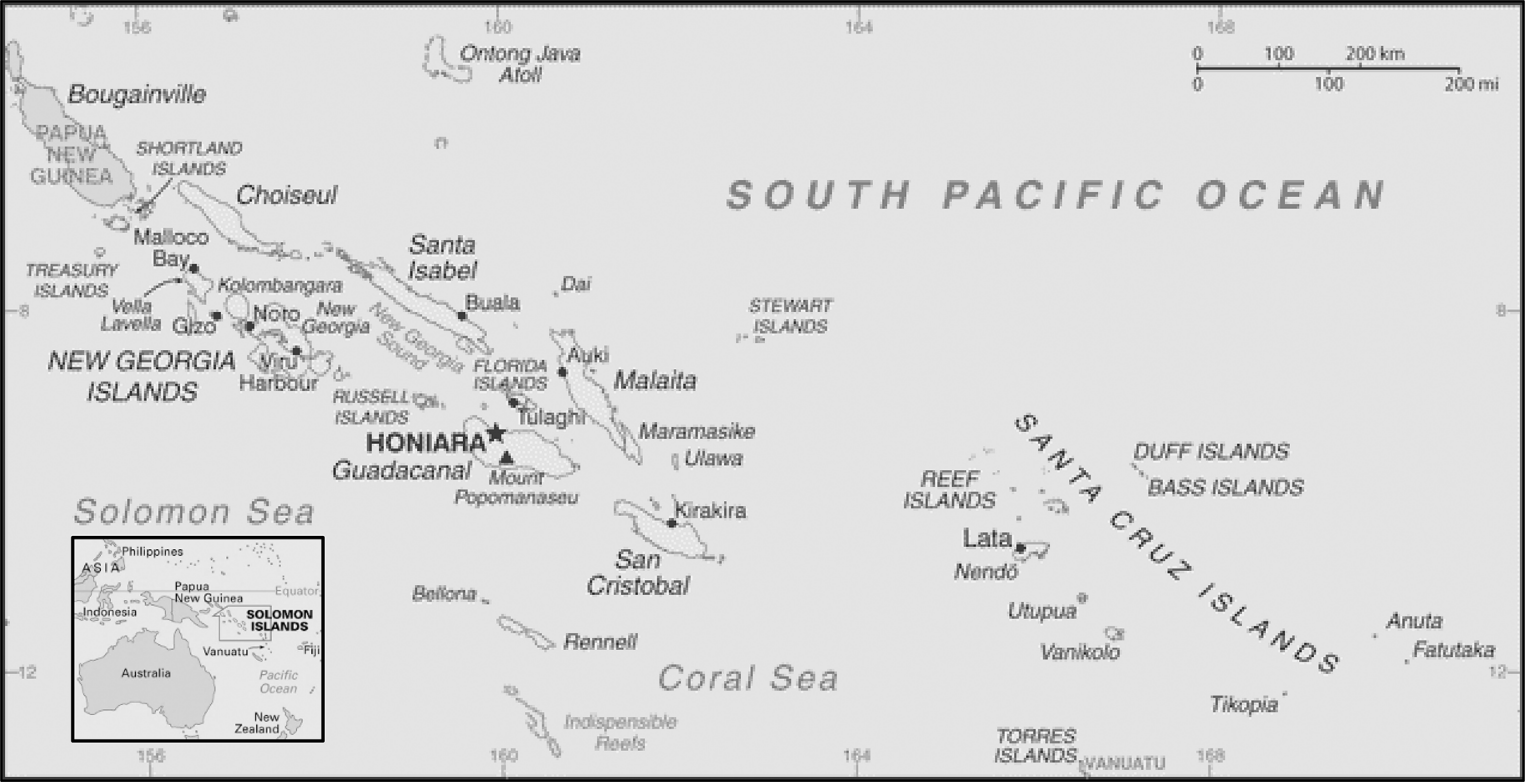
Map of Solomon Islands.

The Solomon Islands (SI), a low-income country ranked 156 of 188 nations in human development [1,2], is located in the south-west Pacific Ocean, approximately 1,800 km north-east of Australia (Fig 1). Disease outbreaks are common in SI with a number of epidemics challenging the country in recent years, including a rubella outbreak in 2012–13 with 13 cases [3]; a rotavirus outbreak in 2014 with over 4,000 cases [4]; a measles outbreak in 2014 affecting 4,563 cases [5]; a Zika virus case in 2015 [6,7]; a cluster of meningococcal disease in 2015 with five paediatric cases [8]; and a dengue virus serotype 3 (DENV-3) outbreak in 2013 with more than 7,000 cases [9].

In March 2011, the SI Ministry of Health and Medical Services (MHMS) established a syndromic surveillance system (SSS) for the early detection of outbreaks and other public health emergencies. The SI-SSS involves the collection of syndrome-based data from ten health facilities (three community clinics; one hospital in the capital city, Honiara; five hospitals in Provincial capitals; and one hospital in a rural setting). Site nurses screen presenting patients for five syndromes (acute fever and rash, acute diarrhoea, influenza-like illness, dengue-like illness and prolonged fever) and report count data to the MHMS on a weekly basis. Data are analysed using an algorithm-based statistical aberration detect approach. Date are analysed at the site and aggregated national levels. Response personnel are alerted when a signal is generated. Surveillance information is disseminated to national stakeholders through a weekly report. In addition, the MHMS has established a mechanism, known as the MHMS Health Emergency Operations Centre (MHEOC), to coordinate outbreak response efforts.

In early-August 2016 [specific date unknown], a sentinel surveillance site in the east of Honiara reported increased numbers of patient presentations with symptoms meeting the dengue-like illness case definition. Subsequently, a large and protracted outbreak of dengue serotype 2 (DENV-2) was identified.

Dengue virus is a tropical mosquito-borne flavivirus, with four antigenically distinct serotypes (DENVs 1–4) [10]. While most dengue infections are asymptomatic, or cause a self-limiting febrile illness of mild to moderate severity, around 5% of infections lead to dengue haemorrhagic fever and dengue shock syndrome [10,11], both of which are serious, potentially fatal illnesses. Over the last 50 years, there has been a 30-fold increase in the reported global incidence of dengue infections [10]. A 2012 study estimated that 3.9 billion people in 128 countries were at risk of dengue infection [12]. Others have estimated there to be 390 million (95% CI: 284–528) dengue infections per year, causing ~1.14 million disability-adjusted life years lost [13,14].

Sporadic dengue outbreaks of varying duration and serotype have been reported in the Pacific islands since the 1950s [15]. Historically, one dengue serotype dominated circulation before being replace by another 3 to 6 years later [16–18]. In the last decade (2008–2018), however, co-circulation of different serotypes has been more common [16]. Since 2008 outbreaks of dengue have been reported in many of the Pacific islands including American Samoa (DENV-4, 2008; DENV-3, 2015; DENV-2, 2017), Cook Islands (DENV-4, 2008), Fiji (DENV-4, 2008; DENV-2, 2012; DENV-2 and DENV-3, 2014; DENV-2, 2015), French Polynesia (DENV-1, 2008; DENV-4, 2009; DENV-1 and DENV-3, 2013; DENV-1, 2015), Kiribati (DENV-4, 2008; DENV-1, 2012; DENV-3, 2014), Marshall Islands (DENV-4, 2011), Micronesia (DENV-4, 2012), Nauru (DENV-4, 2008; DENV-3, 2014; DENV-2, 2017), New Caledonia (DENV-1, 2008; DENV-1 and DENV-3, 2014; DENV-1, 2 and 3, 2017), Niue (DENV-1, 2012), Papua New Guinea (PNG) (DENV-2, 2015), Samoa (DENV-4, 2008; DENV-3, 2015; DENV-2, 2017), Solomon Islands (DENV-3, 2013; DENV-2, 2016), Tonga (DENV-1, 2008; DENV-3, 2014), Tuvalu (DENV-2, 2014), Tokelau (DENV-2, 2015), Vanuatu (DENV-1 and DENV-3, 2014; DENV-2, 2016; DENV-2, 2018), and Wallis and Futuna (DENV-1, 2013 and 2017) [9,16–24]. As at epidemiology week 16/2018 DENV-2 outbreaks were reported to be ongoing in Fiji, New Caledonia, Samoa, Tonga, Vanuatu, New Caledonia and Wallis and Futuna [8,22].

Here we report the epidemiological characteristics of a major DENV-2 outbreak that occurred in SI in 2016–17. We examine the adaptability of syndromic surveillance for enhanced and expanded data collection during a public health emergency and identify and discuss challenges faced in up-scaling surveillance activity in resource-limited contexts.

## Methods

### Data collection

To critique the outbreak, we examined relevant surveillance data (including enhanced and expanded SSS data, outbreak line-listed data, laboratory testing results and patient outcome data) collected by the SI MHMS for the period 7 August 2016 to 30 April 2017. We collected and systematically reviewed relevant documentation (i.e., situation updates, surveillance reports, emails and meeting notes) produced by the MHMS for the same period. To collect data about the challenges operators faced in implementing ehanced and expanded surveillance during the outbreak we interviewed nine key informants including three MHMS staff responsible managing the routine and expanded syndromic systems; three WHO advisors who provided technical support during the outbreak; and the managers of the national environmental health unit responsible for vector control, health promotion unit responsible for community-based risk communication, and the national laboratory which provided laboratory services during the event. Informants were purposefully selected for their knowledge of the system and roles during the outbreak. Seven interviews were conducted face-to-face in Honiara and two by telephone. All interviews were conducted between 5–30 June 2017 using a semi-structured interview format, S1 File. The interview format supported more informal discussion, the emergence of unanticipated insights and allowed the exploration of specific issues that may have not been possible has a more structured approach been taken. Each interview took approximately 1-hour to complete. We applied a content-deductive analysis approach [25] involving the repeated review, systematic organisation and categorisation of qualitative data collected during interviews to identify and characterise emergent themes. Informants comments were recorded in detailed interview notes. A check-back process was undertaken to seek clarification from interviewees where needed. AC conducted the interviews and analysis. Saturation was reached (i.e., new data was redundant of data already collected).

### Case definitions

A suspected dengue case was defined as ‘any person with fever lasting at least two days and two or more of the following: nausea or vomiting; muscle or joint pain; severe headache or pain behind the eyes; rash; spontaneous bleeding’. A confirmed case was ‘any person with laboratory confirmation of dengue infection by rapid diagnostic test (RDT) or reverse transcriptase polymerase chain reaction (RT-PCR)’.

### Diagnostic testing

Serological specimens were collected from a subset of suspected cases and tested in SI using the ‘Dengue Duo’ RDT (Standard Diagnostics Inc., Korea). A RDT was considered positive for dengue if it tested positive for non-structural protein 1 (NS1) or anti-DENV immunoglobulin M (IgM) [10]. No protocol for the selection of cases for testing was developed. The decision to administer a RDT were made by treating health workers based on their availability and a patient presentation.

During the outbreak, two batches of samples were sent to overseas laboratories for confirmatory and further testing by RT-PCR [26–29]for dengue virus. Samples in the second batches was also tested from Zika and chikungunya viruses by RT-PCR [30,31].

### Outbreak management and coordination

A Ministry of Health Emergency Health Operations Centre (MEHOC), comprising senior staff of the SI MHMS, provincial health authorities, local non-government organisations and development partners) was activated to plan, direct and coordinate the national response to the outbreak. Initially, the MEHOC met daily, later meeting weekly. Emergency Operations Centres were activated at the National Referral Hospital (NRH) and in affected provinces to oversee local response activities.

### Statistical analysis

Surveillance data were entered into an Excel (Microsoft Corporation, Redmond, WA) database by MHMS staff over the course of the outbreak. We calculated rates of suspected and confirmed cases using 2016 population projections derived by the SI National Statistics Office based on the 2009 population census [32]. For descriptive analysis, the mean and standard deviation of continuous variables and percentages of categorical variables were calculated. As a proxy for severity, the significance in differences between data collected from hospitalised compared to not hospitalised cases were evaluated using the Chi-squared test. A p-value of <0.05 was considered statistically significant. Odds ratios and their 95% confidence intervals were calculated to assess the strength of relevant associations. Statistical calculations were performed, and figures and tables produced in Excel® (Microsoft Corp., Redmond, WA).

### Ethics

The investigation has the ethical approval of SI Health Research and Ethics Review Board (011/17) and the University of New South Wales Human Research Ethics Committee (HC17238). Written consent was obtained from interviewees. In accordance with the ethical approval, laboratory and patient outcome data collected by the MHMS during the outbreak were provided in fully anonymised form. No new laboratory or patient outcome data were collected.

## Results

### Outbreak identification

**Fig 2:**
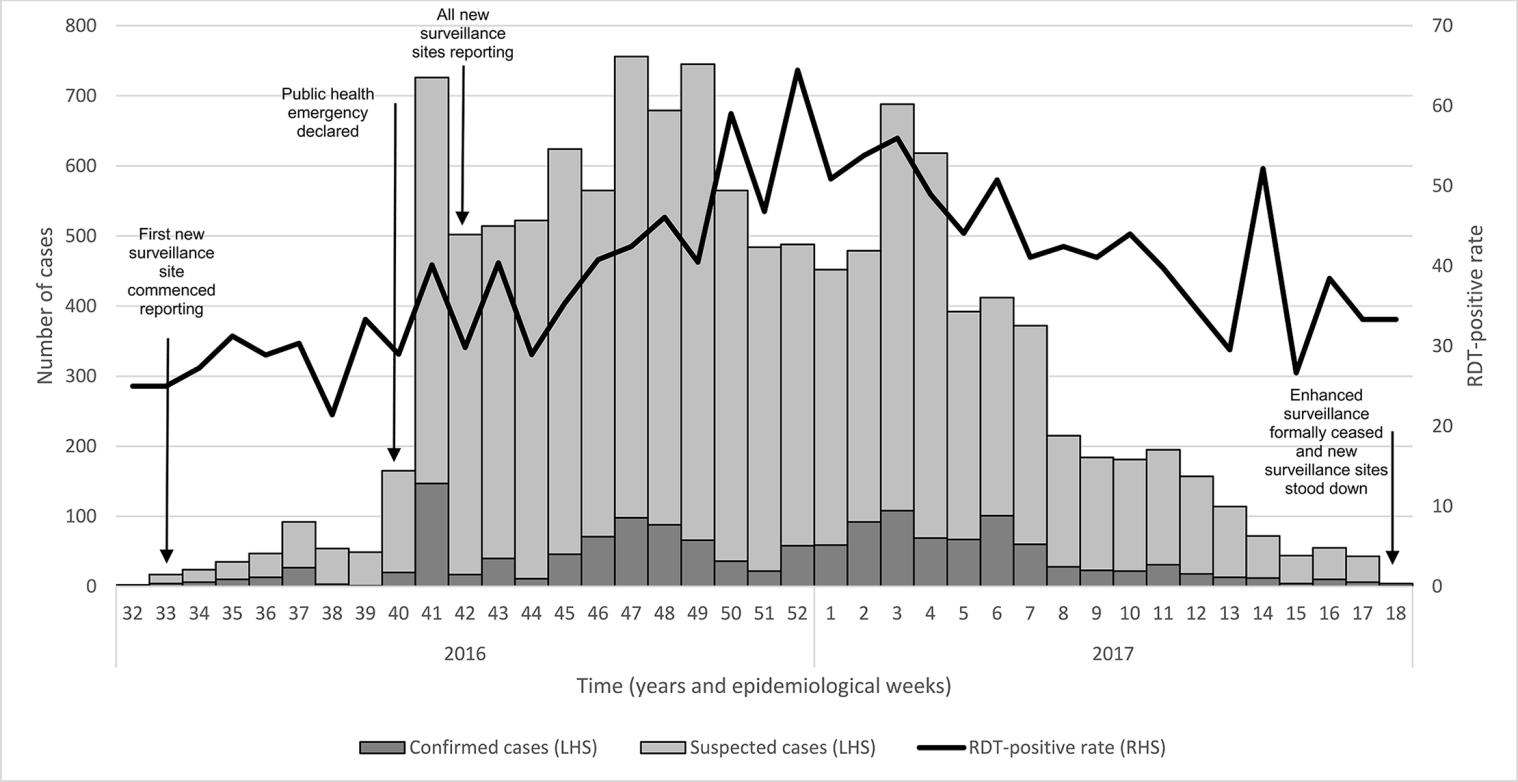
Epidemic curve of the dengue serotype 2 outbreak, Solomon Islands, September 2016-April 2017.

The first suspicion of a dengue outbreak was in early-August 2016 when staff of an established SSS site in eastern Honiara reported unusually high numbers of patients presenting with symptoms consistent with the dengue-like illness case definition. Clinic staff referred an [unknown number] of patients to the NRH for laboratory investigation. The first laboratory-confirmed case of dengue (based on RDT) was on 20 August, in a 62-year-old male resident of Honiara. Over the next month, the MHMS reported an increasing number of patient presentations meeting the ‘dengue-like illness’ case definition in and around Honiara, many of whom tested positive for dengue by RDT (Fig 2). On 8 October 2016, a public health emergency was officially declared in Honiara and the surrounding province of Guadalcanal, and response measures enhanced.

### Epidemiological findings

Between 7 August 2016 and 13 April 2017 (epidemiological weeks 32/2016–18/2017), 12,329 cases meeting the suspected case definition were identified. 3,486 samples (28.3%) were tested by dengue RDT of which 43.3% tested positive. 877 (7.1%) cases were hospitalised and 16 died due to their infection (Case Fatality Rate = 1.3/1,000 suspected cases).

Confirmed cases were identified in nine of the 10 administrative jurisdictions of SI (Table 1). The median attack rate across these jurisdictions was 14.6 (range: 3.6–975.7) per 10,000 population for suspected cases and 2.9 (range: 0.0–159.6) per 10,000 population for confirmed cases. Attack rates were far higher in urban Honiara from where 75.8% of cases were recorded (Table 1).

**Table 1:** Summary epidemiological information about cases of the dengue serotype 2 outbreak, Solomon Islands, 2016–17.

	Population	All cases (suspected and confirmed)	Attack rate (per 10,000 population)	RDT tests conducted	RDT-positive cases	RDT-positivity rate	Hospitalised	Hospitalisation rate
**Sex**								
Male	327,791	6,198	189.1	1,808	820	45.4	473	7.6%
Female	311,627	6,131	196.7	1,678	690	41.1	404	6.6%
**Age**								
<15	259,406	4,339	167.3	1,185	523	44.1	367	8.5%
15–24	119,774	2,956	246.8	619	300	48.5	168	5.7%
25–49	226,696	3,629	160.1	920	372	40.4	258	7.1%
>49	33,542	730	217.6	213	92	43.2	69	9.5%
Unknown	-	675	-	549	223	40.6	15	2.2%
**Province**								
Honiara	82,485	9,342	1,132.6	3,029	1,294	42.7	691	7.4%
Guadalcanal	133,790	2,404	179.7	191	90	47.1	144	6.0%
Malaita	155,457	270	17.4	136	59	43.4	11	4.1%
Temotu	24,278	86	35.4	14	12	85.7	2	2.3%
Renbel	3,823	71	185.7	28	24	85.7	13	18.3%
Choiseul	33,370	59	17.7	54	7	13.0	1	1.7%
Western	92,319	59	6.4	23	15	65.2	5	8.5%
Isabel	32,434	22	6.8	5	5	100.0	4	18.2%
Central	30,837	11	3.6	1	0	0.0	6	54.5%
Makira	50,625	5	1.0	5	4	80.0	0	0.0%
**Total**	**639,418**	**12,329**	**192.8**	**3,486**	**1,510**	**43.3**	**877**	**7.1%**

The outbreak peaked at 756 new suspected cases in a reporting period in late November/early December 2016 before tapering off over the next 4-months (with a slight spike in late January 2017) (Fig 2).

The mean age of cases was 21.8 years (SD = 15.2; range = <1–90 years). Most cases were aged <1–15 year and 25–49 year, representing 35.2% and 29.4% of all suspected cases, respectively. Overall, males and females were equally represented (male:female = 1:1.02). Males (male:female = 1:1.20) were overrepresented in the <15 age group (p,<0.001).

Sixty-one percent of admitted cases were below the age of 25 years. Males were 1.22 (95%CI: 1.04–1.45) times more likely to be hospitalised (p,<0.05) and hospitalised cases were significantly more likely to be confirmed cases (p,<0.05).

Ten of the dengue infection-associated deaths were residents of Honiara, and the other six were from Guadalcanal province. Nine of the 16 deaths were in people older than 50 years, and three were in children less than 15 years of age.

### Clinical findings

Fever, being a cardinal sign of dengue and required to meet the outbreak case definition, was identified in all cases. Gastrointestinal features including anorexia (97.8%), persistent vomiting (95.6%) and abdominal pain (95.4%) were prevalent. Muscle and joint pain (myalgia/arthralgia) was reported by 99.7%, and lethargy by 87.3% of cases. 75.8% of cases presented with a maculopapular rash over the face, upper torso or lower limbs. A significant difference in prevalence of anorexia or nausea; abdominal pain; and rash among hospitalised compared to non-hospitalised cases was found (Table 2). Similarly, confirmation of infection and deaths were both statistically more likely among those hospitalised. It is unknown how many cases experienced headache or retro-orbital pain, generally considered cardinal features of dengue infection, as such data were not collected. Case severity based on the WHO’s severity index was not recorded.

**Table 2:** Clinical features of hospitalised and not hospitalised dengue cases during the 2016–17 dengue serotype 2 outbreak, Solomon Islands.

Characteristics	Hospitalised		Not hospitalised	
	n = 877		n = 11,452	
**Confirmed case**				
**number** ^a^	189	21.6%	1,321	11.5%
**Sex**				
Female	404	46.1%	5,727	50.0%
Male	473	53.9%	5,725	50.0%
**Age**				
Mean (Years)	22.0		22.0	
Standard deviation	17.1		15.1	
Range	<1 yr—86 yrs		<1 yr—90 yrs	
**Age group**				
<15	367	41.8%	3,972	34.7%
15–24	168	19.2%	2,788	24.3%
25–49	258	29.4%	3,371	29.4%
>49	69	7.9%	661	5.8%
Unspecified	15	1.7%	660	5.8%
**Clinical presentation**				
Anorexia or nausea ^a^	100	95.2%	531	98.7%
Arthralgia or myalgia	122	99.2%	736	100.0%
Vomiting	73	97.3%	170	96.6%
Abdominal pain ^a^	66	90.4%	222	97.8%
Mucosal bleeding	45	90.0%	26	81.3%
Lethargy	28	82.4%	50	89.3%
Rash ^a^	18	72.0%	47	90.4%
Tourniquet test	14	66.7%	18	75.0%
**Outcome**				
Death ^a^	7	0.8%	9	0.1%

^a^ A significant difference in proportions between hospitalised and not hospitalised cases (chi-squared, p,<0.05).

### Diagnostic testing

Serological specimens were collected from 3,486 of the 12,329 (28.3%) suspected cases and tested using the ‘Dengue Duo’ RDT. Of these, 1,510 (43.3%) were laboratory-positive. Of the specimens that were laboratory-positive, 1,210 (80.1%) were by detection of NS1 and 446 (29.5%) by detection of anti-DENV IgM antibodies. Both NS1 and anti-DENV IgM were detected in 146 (9.7%) of specimens tested.

Two batches of samples were sent to overseas laboratories for confirmatory and further testing using RT-PCR. On 4 November 2016, a batch of 17 randomly selected dengue RDT positive samples were sent to Pathology Queensland (Australia) and tested for dengue virus. Fourteen (82%) samples returned a positive result for DENV-2 and one for DENV-3. The second batch of 51 samples was sent to the National Centre for Biosecurity and Infectious Disease, Institute of Environmental Science and Research Limited in New Zealand on 7 December 2016 for testing for dengue, Zika and chikungunya viruses. Samples in this batch were chosen based on RDT test results (both positive and negative), age, locality and clinical presentation to aid in the characterisation of the outbreak and to determine if other arbovirus infections were present. DENV-2 was identified in 35 (69%) of samples tested. Testing for other infectious agents, including other dengue serotypes, were negative.

Lack of protocol to guide use of and increased demand for RDTs, particularly during the early stages of the outbreak, led to the exhaustion of test kits.

### Outbreak management and coordination

A dengue triage facility—referred to as the “Dengue Desk”—was established at the NRH to manage the surge in patients seeking care. Nursing staff stationed at the “Dengue Desk” screened all presenting patients using the outbreak case definition and directed those meeting the definition to a designated area of the hospital for specialised treatment. Community and rural health facilities referred suspected dengue cases requiring advanced medical care to the NRH facility.

The MHMS increased health service staffing and reallocated ward space to cater for the surge in suspected cases; sourced additional clinical and diagnostic supplies; revised clinical case management protocols; and provided clinical staff with refresher training in efforts to improve case management. Laboratory tasks were reprioritised to manage demand for RDTs. WHO supported the shipment of specimens to overseas laboratories for confirmatory and further testing.

Community responses included a health promotion campaign delivered through radio, newspapers and door-to-door distribution of risk messages in areas of Honiara where attack rate was highest. The campaign aimed to raise awareness of dengue risk factors, prompt health-care seeking, and influence personal behaviours to minimise mosquito bite risk.

In Honiara, vector control activities aimed to minimise exposure to adult *Aedes* mosquitos by reducing the density and age of adult populations through adulticide space-spraying with deltamethrin and reducing juvenile vector populations through manual removal of habitats during community clean-ups. Vector control efforts in areas outside Honiara were limited to ad hoc community clean-ups. No entomological surveillance data were collected during the outbreak.

It is uncertain to what extent the actions taken reduced the transmission of dengue during this outbreak.

### Enhanced surveillance

In response to the outbreak, the routine SI-SSS was expanded to collect data on patients meeting the outbreak case definitions from an additional 29 health facilities (i.e., a total of 39 reporting sites, including 30 community health facility and nine hospital sites). Nurses at the 39 sites were instructed to apply the case definition to all presenting patients and to record and report demographic characteristics (name, age, sex, place of residence), symptom (fever, rash, mucosal bleeding anorexia, arthralgia, abdominal pain, persistent vomiting, lethargy, fluid retention, liver enlargement, tourniquet test), date of symptom onset, rapid diagnostic test result, and hospitalisation status data for patients that met the definition on a provided form. The first new sentinel sites began reporting data in epidemiological week 33/2016 with others contributing by epidemiological week 42/2016.

Data were transferred from surveillance sites to the Honiara-based MHMS surveillance unit on a weekly basis by various means, including collection of reporting forms by MHMS staff (number of surveillance sites = 17, 44%), hand delivery of forms to MHMS staff (number of surveillance sites = 12, 31%), by email (number of surveillance sites = 4, 10%), and verbally by telephone (number of surveillance sites = 6, 15%). MHMS surveillance staff manually entered data received into an Excel^®^ database for analysis. The resulting information was presented in a weekly report sent to ~120 recipients including staff of national and provincial health services, other government departments, development partners and relevant SI-based non-government organisations. The MEHOC used the information provided to monitor the temporo-spatial evolution of the outbreak and target response efforts.

While enhanced surveillance was ceased on 13 April, 2017 health facilities were instructed to remain vigilant for patients meeting the case definition and to notify the MHMS of suspected cases immediately.

### Qualitative interviews

Key informants reported that enhancing and expanding surveillance placed considerable strain on what they, and others [33–35], acknowledge is a vulnerable health system. Surveillance staff identified that the added data collection and processing resulting from enhanced and expanded surveillance activities quickly overwhelmed the capacity of the MHMS’s surveillance unit. The burden placed on the unit’s staff was amplified by the need to manually cross-reference sites’ reported data to avoid double counting cases that sought care at multiple facilities. Computer entry of received data was labour intensive, time-consuming and difficult to maintain as case numbers increased, even when additional data entry staff were engaged. “We engaged more staff, but still we were overwhelmed”, a surveillance officer said.

MHMS surveillance staff found inconsistency between suspected dengue case count and line-listed data, prompting concern about data consistency. A surveillance staff member commented, “…it was not possible to effectively integrate the data we were collecting about dengue patients with the ongoing syndromic surveillance data. “Dengue surveillance, combined with all the other things we needed to do was exhausting”, a surveillance officer commented.

Surveillance officers noted that while expanding the SSS improved coverage the MHMS’s capacity to support staff new to surveillance was inadequate, a factor alluded to as having contributed to compromised system stability and data integrity.

Interviewees reported that data quality monitoring was not undertaken during the outbreak and as such it was unclear how rigorously the case definitions were applied. The requirement for nurses to collect line-listed data in addition to syndromic count data was said to be cumbersome and confusing for some, raising suspicion that adherence to data collection protocols was compromised.

Surveillance data were reported weekly which, in the opinion of those responsible for outbreak response activities, was insufficient to guide targeted response actions. Two interviewees note that the lack of case geo-location data further restricted ability to target interventions. “We wanted to target households but had to go to the local clinic to ask nurses which households we should visit; they were often too busy to help us” one interviewee reported.

Data on the core syndromes (i.e., acute diarrhoea, bloody diarrhoea, influenza-like illness and prolonged fever) continued to be collected at the ten established sentinel surveillance sites in parallel to dengue surveillance for the duration of the outbreak. Maintaining both systems and identifying resources to respond to surveillance signals for syndromes other than dengue was reported to be problematic given the focus on the dengue response.

The overwhelming volume of work and the protracted nature of the outbreak contributed to staff fatigue which, as the event continued, led to absenteeism and apathy towards staffs’ data collection and reporting responsibilities.

## Discussion

We report the investigation, findings and response to the largest and longest-running dengue outbreak recorded in SI. As information on past dengue outbreaks in the Pacific islands is incomplete comparing the size of this outbreak with others was not possible, however, given the scale and duration of this dengue outbreak it appears to have been one of the most significant to have occurred in the region.

While other authors have characterised dengue outbreaks to affect Pacific islands [9,16,17,19–24,36–38] we understand that our paper is the first to explore the adaptability of and challenges in enhancing and expanding a routine SSS to meet increased information demands during a public health emergency in a small island developing state context. The surveillance response to the outbreak highlighted several health system issues.

As dengue is a serotype immunising infection, it is possible that younger Solomon Islanders were immunologically naive to DENV-2 and as such vulnerable to infection. Co-circulation of DENV-2 and DENV-3 was evident based on findings from the first batch of referred samples, while in the second batch only DENV-2 was detected. Given DENV-3 was the only serotype identified in samples tested during the 2013 dengue outbreak [9], these laboratory results may indicate replacement of DENV-3 as the predominant circulating dengue serotype in SIs, a phenomenon observed by Li et al. (2010) as having occurred in the Pacific islands in the past.

The high concentration of reported cases in Honiara (Attack Rate: 1,132.6/10,000 population) is likely due to a combination of factors including better access to health care and hence case detection and reporting, and rapid urbanisation in recent years (urban growth rate of 4.8% compared to national population growth rate of 2.3% [39]) putting pressures on housing, water, waste management and environmental hygiene; all contributing to the creation of habitats conducive to *Aedes* mosquito breeding. As noted by Nogareda et al. (2013) and Shortus et al. (2014), knowledge of *Aedes* mosquito habitats distribution in SI is limited with past entomological studies only having investigated (and identified) the species in Honiara [9,40]. Unfortunately, due to a lack of in-country capacity and resources vector surveillance was not conducted during the outbreak. Vector surveillance data would have complemented disease surveillance data and informed better analysis and targeted vector control operations. This highlights the importance of routine surveillance of *Aedes* populations, as well as conducting pre- and post-control surveys, in populated areas at elevated risk of outbreaks.

We note that the rapid increase in case detection coincided with the expansion of the surveillance system and the declaration of a public health emergency (Fig 2). These factors likely represent the rapid increase in cases detected from epidemiological week 40/2016. As it is difficult to quantify what proportion of the increase was due to excess disease occurrence and what was a product of expanded surveillance activities, caution should be shown in assuming that the increase mirrors the outbreak’s evolution. A greater utility is to monitor trends from when the enhanced and expanded surveillance system were operational (i.e., from epidemiological week 41/2016 onward). Similarly, variances in the coverage of the surveillance system, of the populations’ health-seeking behaviours, and the availability of dengue RDTs across the country likely introduces a measurement bias in the data collected which, together with small data counts in some settings, means results should be interpreted with caution.

The dengue outbreak placed considerable strain on what is a vulnerable health system requiring it to be proactive and adapt rapidly to increased demands. Of note was the MHMS’s ability to scale-up the routine early warning SSS in response to the outbreak and thereby produce relatively timely information on which the MEHOC relied. This action demonstrates the flexibility of the SI-SSS and provides an example of the flexibility of syndromic surveillance for outbreak detection and monitoring in low resourced contexts more broadly. Despite this, expansion of the system was not without its challenges.

We found that the MHMS surveillance unit had limited capacity to support new sites implement surveillance practices. Given no data quality monitoring was conducted it is unclear how rigorously the outbreak case definitions were applied, or cases’ signs and symptom data collected. The potential of poor data quality somewhat undermines the reliability of evidence produced by the system and hence information on which decision makers rely. This is particularly pertinent for settings where decision makers are dependent on single indicator-based surveillance strategy for timely information, as is the case in SI. To ensure system integrity during public health emergencies, preparedness activities must include strategies to support the quality of surveillance data. Strategies should include rigorous screening of proposed new sentinel sites to ensure they have the capacity to perform required tasks; provision of resources required to perform surveillance duties; development of tools that streamline the capture of surveillance data, including their extraction from routine clinical records where feasible; support of staff new to early warning surveillance; mentoring and supervision of front-line nurses; establishment of protocols for inter-jurisdictional data sharing; development and testing of plans for system expansion; and periodic monitoring and feedback of data accuracy and utilisation.

Focused environmental interventions, such as community-based environmental management to disturb potential vector breeding sites, are proven to provide protection during dengue outbreaks [41]. The suggestion that the frequency at which surveillance data were collected, analysed and reported was insufficient to guide such efforts during the outbreak is noteworthy as it relates to the core purpose of the system, that being timely information generation to inform prompt responses. Analysis of the value and feasibility of more frequent data reporting is needed. In the short-medium term, international development partners have a pivotal role to play in both supporting system development and through supplementation of resources (including human resources) in response to surges in demand.

The increased volume of data collected during the outbreak was found to overwhelm the capacity of the MHMS’s surveillance unit. The data collection, reporting and collation process may have been streamlined through the adoption of mobile technology. Mobile technology has been successfully used as part of early warning surveillance in several developing settings [42–52]. Owing to the similarities SI shares with neighbouring Fiji and PNG, special attention should be paid to the work of the WHO (in Fiji) and Rosewell et al. (2017) (in PNG) who report national experiences using mobile devices and Global Information Systems for outbreak and infectious-disease surveillance.

From a health systems perspective, it is essential that the introduction of new technology to enhance early warning surveillance complement efforts to improve broader health information management, including interoperability with existing health information system platforms.

For the duration of the outbreak, data collection for other syndromes continued at the ten established sentinel sites in parallel to enhanced dengue surveillance. Maintaining both systems and identifying resources to respond to surveillance signals for syndromes other than dengue-like illness was a challenge. In SI this observation is not unique with past public health emergencies quickly consuming available resources [4,9]. Building capacity to maintain the SI-SSS’s primary function (i.e., rapid detection of outbreak-prone diseases) while responding to emergencies is needed. This will require building both the capabilities and efficiency of teams at the national and subnational levels responsible for surveillance and response to better manage surges in demand. An element of redundancy should be expected. Building capacity to identify emergent disease threats at all times (including during protracted public health emergencies, such as the DENV outbreak) will ensure SI is complying with its surveillance-related International Health Regulation core capacity obligations [53].

The increased demand for RDT led to the rapid exhaustion of test kits. Dengue RDTs are useful for identifying a dengue outbreak but are less useful in individual case management in clinical settings. The sensitivity and specificity of dengue RDTs are variable depending on factors including DENV serotype, date of collection, the skill of technician and history of previous DENV infections in individuals [54]. The lack of guideline for the appropriate use of dengue RDTs during an outbreak likely contribute to their inappropriate use and stock depletion. Further guidance on the use of dengue RDTs during outbreaks will support their prudent use for public health purposes.

Addressing risk posed by DENV in SI will take a multi-strategy approach and require action from parties broader than just the health sector, both in preparation and response. In terms of preventing and preparing for future outbreaks, priority actions include: reducing sites conducive to *Aedes* mosquito breeding and improving water and sanitation infrastructure; ensuring emergency operations plans are functional and integrated across all levels and agencies of government; and that the national early warning surveillance system has the resources needed and procedures in place for prompt identification, verification and investigation of surveillance signals, as well as the capability to scale-up when required. With regard to response, having staff and systems in place to support event management processes (including risk assessment) and the capacity available to implement the suite of actions required to effectively manage dengue outbreaks is required. These measures include enhancement of clinical case management; active and passive vector control; risk communication; epidemiological case identification, investigations and mapping; and specimen collection and laboratory confirmation [10,55–57].

## Conclusions

The SI remains vulnerable to outbreaks of dengue and other outbreak-prone communicable diseases. This case study demonstrates that SSSs can–relatively easily–be expanded and enhanced during an outbreak situation in a low-resource setting, however, issues of data quality and timeliness must be managed. During prolonged outbreaks fatigue amongst surveillance staff should be expected. Providing daily, rather than weekly, surveillance data on suspected dengue cases would be more useful for response planning and may lead to better public health outcomes. Incorporating mobile technology into surveillance practice may aide more timely data capture which, coupled with geo-coding ability and automated analysis, will likely enhance outbreak informatics and target control activities. Further efforts to determine the extent to which the response to the DENV-2 emergency reduced and prevented transmission is required.

## Supporting information

S1 File Key informant interview data collection tool. (PDF)
