# Supplementary material for: Enhanced surveillance during a public health emergency in a resource-limited setting: Experience from a large dengue outbreak in Solomon Islands, 2016-17
Source: PLoS One. 2018 Jun 7;13(6):e0198487. doi: 10.1371/journal.pone.0198487 (PMC5991673; doi:10.1371/journal.pone.0198487)
Supplement: S1 File — (PDF) [file pone.0198487.s001.pdf]

Solomon Island dengue outbreak and response review í stakeholder interview data collection tool

1. Administration

Consent to participate in interview?

Yes O No O

Name ______________________________

Date ______________________________

Location ______________________________

2. What was your role during the dengue outbreak? Specifically, what tasks were you responsible for?

____

____

____

____

3. In your view, how did the SI-SSS perform during the dengue outbreak? What were the major challenges faced and how, from your perspective, did the MHMS respond?

____

____

____

____

4. The system expanded to collect data from more sites very quickly. How did this go? What challenged did you see/experience? How were these managed?

5. Health facilities experienced a large increase in patients seeking care during the outbreak. What impact did this have on the health system, and how did the MHMS/hospital actions manage these?

6. Thinking specifically about your role during the outbreak, what challenges did you (and your team) face? What caused these challenges? How were they managed?

7. How did you use the information produced by the enhanced outbreak surveillance system? Did the information provided meet your information needs at the time? If not, why not?

8. In your view, how reliable was the data that was being produced? What enhanced or undermined the information produced?

9. Is there anything else you would like to add?

____

____

____

____

Thank you for your participation.
